# On-chip complex refractive index detection at multiple wavelengths for selective sensing

**DOI:** 10.1038/s41598-022-13033-3

**Published:** 2022-06-04

**Authors:** Raghi S. El Shamy, Mohamed A. Swillam, Xun Li

**Affiliations:** 1 Faculty of Engineering, Department of Electrical and Computer Engineering, McMaster University, Hamilton, ON L8S 4L8 Canada; 2 Department of Physics, School of Science and Engineering, The American University in Cairo, New Cairo, 11835 Egypt

**Keywords:** Integrated optics, Optical sensors, Microresonators, Silicon photonics

## Abstract

In this work we propose a method for on-chip detection of the complex refractive index of the sensing medium at multiple wavelengths for selective sensing. For the optical sensor to be selective, i.e. able to determine the substance present in the medium, either surface functionalization or absorption spectroscopy is often used. Surface functionalization is a complex process and is mainly limited to biological media. On the other hand, absorption spectroscopy is not suitable for on-chip sensing with micrometer dimensions as this will result in poor sensitivity, especially when working far from the substance absorption peaks. Here, we detect the dispersion of both the real n and imaginary k parts of the refractive index which are unique for each substance. This is done using a single micro-ring resonator (MRR) that exhibits multiple resonances over the operating wavelength range. The real and imaginary parts of the medium refractive index are determined at each resonance using the resonance wavelength and the absorption coefficient, respectively. In addition, using this technique the concentration composition of a multi-element medium can be determined by solving a system of linear equations that corresponds to the different wavelengths (resonances). We designed a silicon-on-insulator (SOI) ring-resonator operating in the near-infrared region from λ = 1.46 µm to λ = 1.6 µm. The ring exhibits 11 resonances over the 140 nm operating wavelength range where the corresponding medium refractive index is obtained. This design can detect four different substances namely, methanol, ethanol, propanol, and water. An average error of less than 0.0047% and 1.65% in the detection of the real and imaginary parts, respectively were obtained. Finally, the concentration composition of different multi-element media were successfully determined using the least square method with 97.4% detection accuracy.

## Introduction

Chemical and biological detection and monitoring of various types of molecules is an essential task in many fields. Healthcare, medical diagnosis, food industry, environmental monitoring, safety, and industrial process control are all areas where different substances sensing is needed^1^. In general, optical sensors exhibit higher sensitivity, wider dynamic range, and immunity to electromagnetic interferences over their electrical counterparts^2^. Moreover, integrated optical sensors offer mass scale hence low-cost fabrication and high throughput sensing using multiplexed sensor array^3,4^. In addition, co-integration with electrical functions is possible resulting in compact portable devices capable of real-time detection. Thus, integrated optical sensors are a promising platform for Lab-on-Chip technology^4^.

There are mainly two techniques in integrated label-free optical sensing namely refractive index (RI) and absorption sensing^3,5^. RI sensor detects the real part *n* of the medium (analyte) refractive index^6–19^. RI sensors are suitable for small volume samples as *n* is related to concentration, not the total sample mass^3^. Hence, they are a good candidate for integrated on-chip optical sensing. Another advantage of RI sensors is that small changes in *n* over small distances can lead to a large change in the propagating wave phase reaching very high sensitivities through different optical devices. Mach–Zehnder Interferometer (MZI)^7–10^ and micro-resonators^11–15^ are the most widely used optical devices for RI sensing in addition to sensors using surface plasmon polariton (SPP) waves^2,16–19^. However, RI sensors are not selective, i.e. they determine only the value of the refractive index *n* but cannot determine the substance that resulted in this refractive index. At a certain wavelength, different substances with different concentrations can result in the same *n*. Moreover, in the case of a multiple element medium, the sensor will determine the resultant *n* and not the elements composition of this medium. Thus, selectivity is one of the main issues regarding RI sensors. One widely used technique to solve this problem in bio-sensing is surface functionalization^4,20–24^. In surface functionalization, the surface of the sensing waveguide is coated with specific molecules called binder or capture molecules and immobilized through a certain process. The immobilized molecules selectively capture the analyte molecules to be detected from the whole sample. Although this technique is selective and widely used in bio-sensing however, it involves many lengthy processes to functionalize the sensor surface with the capture molecules. In addition, a washing step after sensor exposure to the sample is needed to make sure that only the analyte of interest is present in the sensor medium. Moreover, this surface functionalization process may be challenging or even not possible on some materials. Also, as mentioned this technique is used only with biological samples. Furthermore, for multi-element detection, multiple output signals need to be detected which complicates the sensor circuitry.

On the other hand, absorption sensors which detect the imaginary part *k* of the medium refractive index can be selective^25–28^. The absorption spectrum of the sample is measured and then used to specify the different substances present in the sample through their absorption fingerprints^25^. However, the sensitivity of this technique depends on the sample volume because larger interaction lengths between the light and sample will result in higher light absorption hence higher sensitivity^28^. Consequently, integrated waveguide based absorption sensors with micrometer dimensions suffer from poor sensitivity. To overcome this problem high-quality factor (Q) micro-cavities were proposed to enhance the interaction length between the mode field and the analyte^26–28^. In high-Q cavities, the light circulates multiple times in the cavity and hence the effective optical path length can be increased significantly from micrometer to millimeter range^26–28^. In^28^ authors used micro-ring resonators with Q > 100,000 to enhance the interaction length and hence sensitivity for on-chip absorption spectroscopy. The absorption spectrum of N-methylaniline was successfully obtained in this work. In^29^ authors were detecting the complex refractive index of the sensing medium by measuring changes in the resonance wavelength and the resonance linewidth of a photonic crystal micro-cavity from which they detect the complex refractive index of the medium. Using this technique they were able to determine the concentration composition of a ternary mixture.

In this work we present a method for on-chip complex refractive index detection for selective sensing. Using our proposed method both real *n* and imaginary *k* parts of the medium refractive index are determined at multiple wavelengths over a certain wavelength range. This is done simply using a single micro-ring resonator (MRR) with multiple resonances along the wavelength range of operation where both *n* and *k* are determined at each resonance. In this case, the medium complex refractive index dependence with wavelength, i.e. medium dispersion, is determined rather than just a single value at a single wavelength. While at single wavelength different substances can lead to the same *n* or *k*, the refractive index dispersion is specific for each substance. Fitting these discrete *n* and *k* data with wavelength can then specify the medium substance. On the other hand, the mixture composition of a multi-element medium can also be determined by solving a system of linear equations corresponding to the different wavelengths. This method is similar to the one proposed in^28^ however we here determine the complex refractive index while they were only interested in the detection of the medium absorption coefficient *α*. A ring resonator using the widespread silicon-on-insulator (SOI) technology, working in the near-infrared region around λ = 1.55 µm is implemented. Our designed resonator can selectively detect methanol, ethanol, propanol, and water in a single element medium with high accuracy. It is also able to determine the concentration composition of multi-element media. In general, many sources of error can lead to inaccuracy in the detected concentrations for instance, temperature fluctuations. Determining both *n* and *k* at multiple wavelengths increase the detection accuracy. This technique can be used with any chemical or biological substance. It is also possible that this technique can be used to overcome the need for complex surface functionalization process. It is efficient even if the sample substance exhibit low absorption as both *n* and *k* are detected simultaneously. Finally, a multi-element sample is detected through a single output.

## Complex refractive index detection

### Methods

To determine the medium complex refractive index *n* + *jk* over a range of wavelengths, a MRR with multiple resonances along this range is designed. Changing the real part of the medium refractive index results in a shift in the resonance wavelength *λ*_*res*_. While changing the imaginary part of the medium index results in a change in the cavity losses and accordingly the field attenuation coefficient *ɑ*. Hence, at each resonance the corresponding *n*_*i*_ and *k*_*i*_ of the medium are determined, where *i* = 1 − *N* is the resonance index and *N* the number of resonances in the measured wavelength range.

The transmission spectrum of the MRR with lossless coupling (*r*^*2*^ + *t*^*2*^ = 1) is given by^28^:

1 $$T(\theta ) = \frac{{a^{2} + r^{2} - 2ar\cos (\theta )}}{{1 + a^{2} r^{2} - 2ar\cos (\theta )}}$$

2a $$a = e^{ - \alpha L}$$

and

2b $$\theta = \beta L = \frac{2\pi }{\lambda }n_{eff} L$$

3a $$\alpha = \alpha_{{\text{int}}} + \alpha_{abs}$$

and

3b $$\alpha_{abs} = \frac{2\pi }{\lambda }k_{eff}$$

where *ɑ* the attenuation factor and *α* the field loss coefficient. The field loss coefficient constitutes of two parts the intrinsic loss *α*_*int*_, such as waveguide surface roughness and bend loss, and medium absorption loss *α*_*abs*_. *r* the forward-coupling coefficient, the fraction of field that transmits to the straight waveguide while *t* the cross-coupling coefficient, the fraction of field that is coupled to the ring cavity. *θ* the round trip phase where *L* the cavity length *L* = *2πR*, *β* the mode propagation constant, *n*_*eff*_ the mode effective index, and *R* the ring radius.

A reference medium with known refractive index *n*_*ref*_ and *k*_*ref*_ is used from which we calculate the change in the resonance wavelength and the change in the loss (*Δλ*_*re*s_ and *Δα*). We first fit each resonance of the MRR spectrum both of the reference and the sensing medium with wavelength using Eq. (1) to obtain the resonant wavelengths *λ*_*res,i*_ and the attenuation factors *ɑ*_*i*_, from which the absorption loss coefficient *α*_*i*_ is calculated using Eq. (2a). When doing the fitting we expand the cosine term in Eq. (1) around the resonant wavelength *λ*_*res*_ such that the fitting parameters are only *λ*_*res,i*_ and *ɑ*_*i*_ while the rest of the terms are known from the design, i.e. *r* and *L* = *2πR*, see Eq. (4). Note that, the group index *n*_*g*_ in Eq. (4) can be easily calculated from the measured free spectral range (FSR) and resonance spacing using Eq. (5).

4 $$\cos (\theta ) = \cos (2\pi Ln_{eff} /\lambda ) \simeq 1 - \frac{{2\pi^{2} L^{2} n_{g}^{2} }}{{\lambda_{res}^{4} }}(\lambda - \lambda_{res} )^{2} + \frac{{2\pi^{4} L^{4} n_{g}^{4} }}{{3\lambda_{res}^{8} }}(\lambda - \lambda_{res} )^{4}$$

5 $$FSR = \frac{{\lambda^{2} }}{{n_{g} L}}$$

Then, the change in the mode effective index of the sensing medium from the reference, both the real *Δn*_*eff,i*_ and the imaginary *Δk*_*eff,i*_ part, are calculated from the resonance wavelength shift *Δλ*_*res,i*_ (resonance condition) and the change in the loss coefficient *Δα*_*i*_, respectively using Eqs. (6a) and (6b). This method is similar to the one used in^28^. However, they were fitting Eq. (1) with *ɑ*_*i*_ as they were interested only in the detection of the medium absorption coefficient *α*.

6a $$\Delta n_{eff} = n_{eff,sens} - n_{eff,ref} = \frac{{n_{eff} }}{{\lambda_{res} }}\Delta \lambda_{res}$$

6b $$\Delta k_{eff} = k_{eff,sens} - k_{eff,ref} = \frac{\lambda }{2\pi }\Delta \alpha$$

Next, we calculate the medium index change *Δn*_*med,i*_ and *Δk*_*med,i*_ from *Δn*_*eff,i*_ and *Δk*_*eff,i*_ as follows:

7a $$\Delta n_{eff} = S_{wg} \Delta n_{med}$$

7b $$\Delta k_{eff} = S_{wg} \Delta k_{med}$$

7c $$S_{wg} = \frac{{dn_{eff} }}{{dn_{med} }} = \frac{{dk_{eff} }}{{dk_{med} }}$$

where *S*_*wg*_ the waveguide sensitivity. Finally, the absolute complex refractive index of the sensing medium is determined using the known reference medium index as:

8a $$n_{sens\_med} = n_{ref\_med} + \Delta n_{med}$$

8b $$k_{sens\_med} = k_{ref\_med} + \Delta k_{med}$$

### MRR sensor design

In this section, we design the MRR for the complex index detection, namely the waveguide width *w* and the ring radius *R*. The resonator is based on the standard SOI technology with thickness h = 220 nm and is operating in the near-infrared region with 140 nm bandwidth from 1.46 to 1.6 µm. The main performance parameters of the ring resonator sensor can be derived from the relations of Eqs. (1–3). The wavelength sensitivity *S*_*n*_, the attenuation factor sensitivity *S*_*k*_, the full width half maximum *δλ*_*FWHM*_, the minimum/maximum detectable real refractive index change *Δn*_*min*_*/Δn*_*max*_, and the minimum/maximum detectable imaginary refractive index change *Δk*_*min*_*/Δk*_*max*_ are given by:

9a $$S_{n} = \frac{d\lambda }{{dn}} = \frac{\lambda }{{n_{eff} }}S_{wg}$$

9b $$S_{k} = \frac{da}{{dk}} = - aL\frac{2\pi }{\lambda }S_{wg}$$

10 $$\delta \lambda_{FWHM} = FSR\frac{{1 - a^{2} }}{\pi a}$$

11a $$\Delta n_{\min } = \frac{{\Delta \lambda_{\min } }}{{S_{n} }} = \frac{{n_{eff} }}{{\lambda S_{wg} }}\Delta \lambda_{\min }$$

11b $$\Delta n_{\max } = \frac{FSR}{{S_{n} }} = \frac{\lambda }{{S_{wg} L}}$$

12a $$\Delta k_{\min } = \frac{1}{{2S_{n} }}\frac{a}{{1 + a^{2} }}\Delta \lambda_{min} = \frac{1}{2}\frac{a}{{1 + a^{2} }}\Delta n_{min}$$

12b $$\Delta k_{\max } = \frac{1}{2\pi }\left. \frac{da}{a} \right|_{\max } \Delta n_{\max }$$

**Figure 1:**
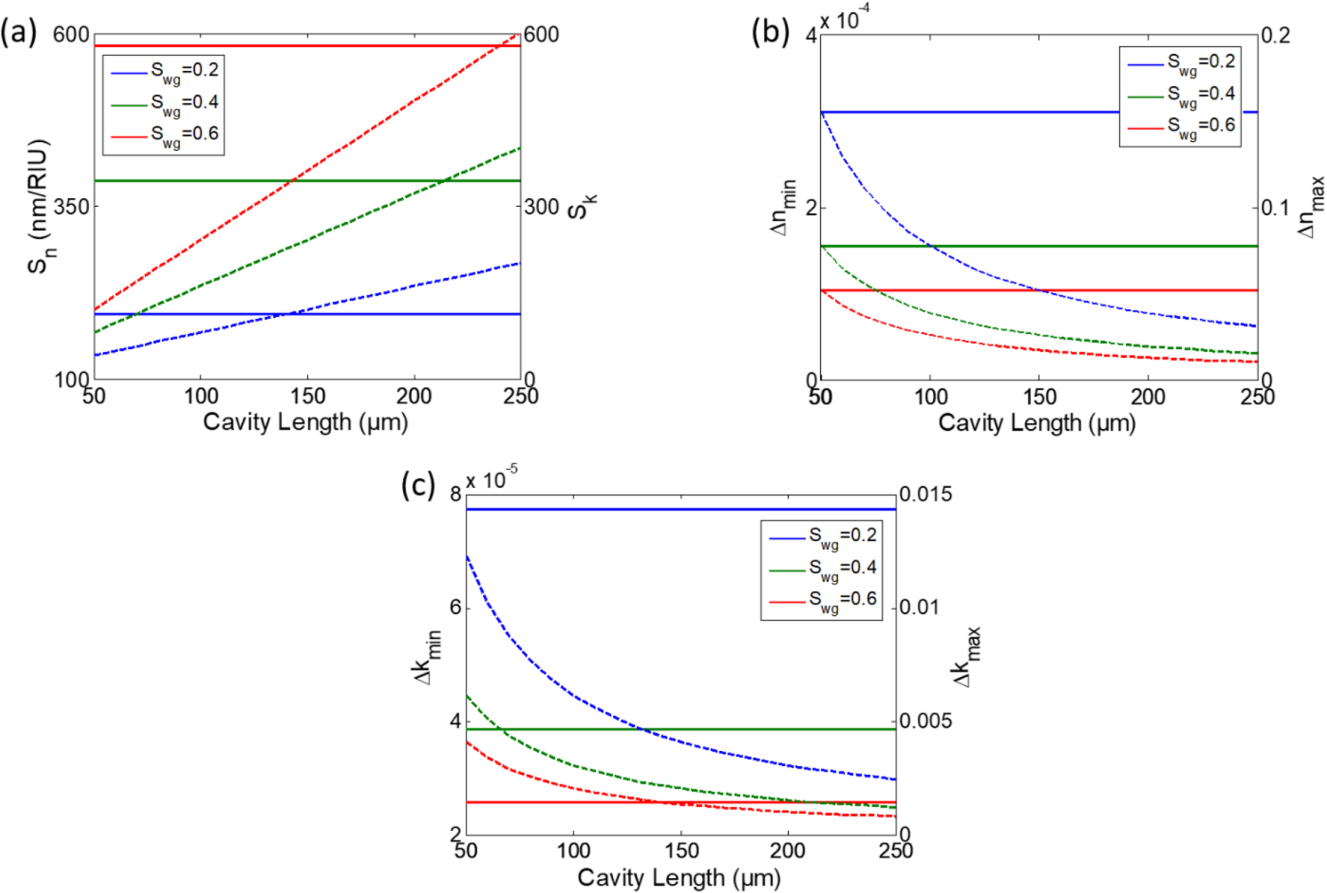
(**a**) *S*_*n*_ "solid"and *S*_*k*_ "dotted", (**b**) *Δn*_*min*_ "solid" and *Δn*_*max*_ "dotted", (**c**) *Δk*_*min*_ "solid" and *Δk*_*max*_ "dotted" versus cavity length at different S_wg_.

Figure 1 shows the dependence of the different MRR sensor performance parameters on the ring design parameters, namely the S_wg_ (determined by the waveguide width) and the *L*. The ring radius *R* determines the *FSR* of the cavity Eq. (5). Hence, increasing *R* will reduce the *FSR*, i.e. there will be more resonances in the operating wavelength range, accordingly more number of points in the detected *n* and *k*. However, reducing the *FSR* will limit the maximum refractive index change that can be detected, see Eq. (11b) and Fig. 1b,c. For a specific waveguide width there is a minimum radius *R*_*min*_ to ensure low bend loss. So to be able to detect a wide range of *Δn* we work on *R* ≈ *R*_*min*_. Note that, as the waveguide width decreases as this *R*_*min*_ increases.

**Figure 2:**
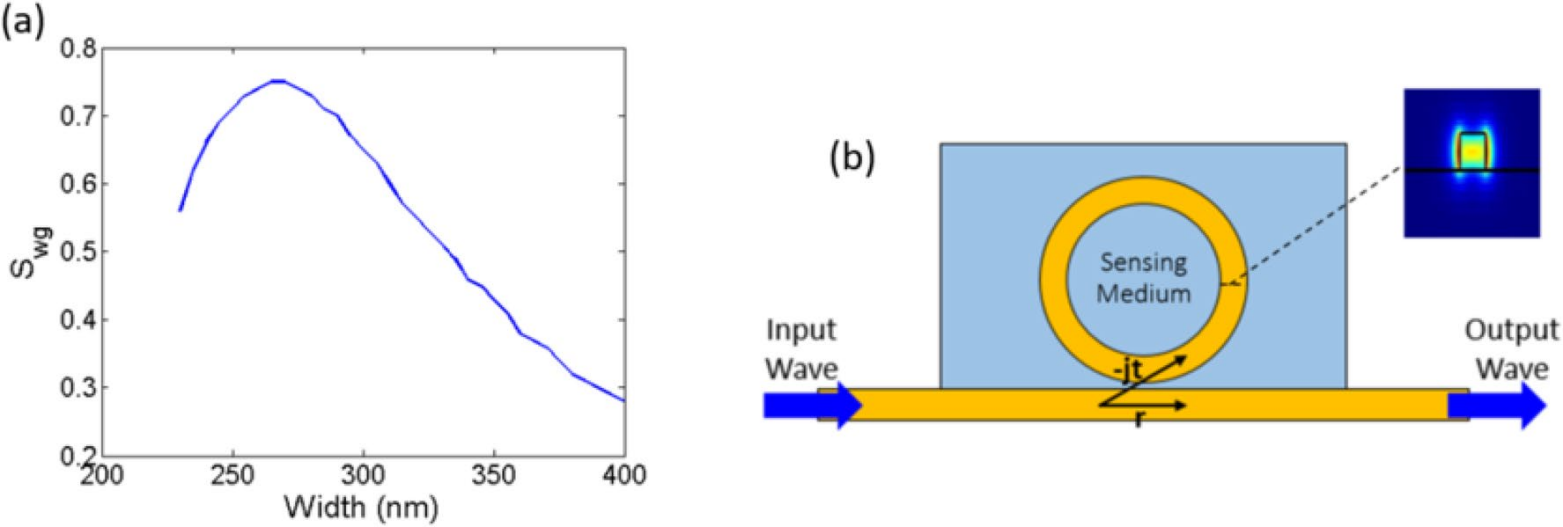
(**a**) S_wg_ versus waveguide width w at λ = 1.55 µm. (**b**) Schematic of the MRR sensor; inset: electric field intensity profile of the used SOI waveguide (w = 320 nm) fundamental quasi-TE mode at λ = 1.55 µm.

On the other hand, the width of the waveguide determines the waveguide sensitivity *S*_*wg*_. We need to maximize *S*_*wg*_ to maximize the sensor sensitivity both to changes in *n* and *k* as Eqs. (9a, 9b) and Fig. 1a depict. This enhances the sensor performance as it will minimize the lowest detectable *Δn*_*min*_ and *Δk*_*min*_ according to Eqs. (11a) and (12a). Figure 2a shows the *S*_*wg*_ of the SOI waveguide fundamental quasi-transverse electric (TE) mode versus the waveguide width w at λ = 1.55 µm, where we can see that there is an optimum width *w*_*opt*_ = 270 nm. For *w* = 270 nm, *R*_*min*_ = 32 µm for *ɑ*_*bend*_ > 0.99, where *ɑ*_*bend*_ field attenuation coefficient due to bend loss. In this case, the maximum *Δn* that can be detected is *dn*_*max*_ = 0.01. Also note that, both *Δk*_*min*_ and *Δk*_*max*_ are proportional to *Δn*_*min*_ and *Δn*_*max*_, respectively. Thus, optimizing the real part parameters will also optimize the imaginary part parameters.

Here we design a MRR to detect four different elements namely, methanol, ethanol, propanol, and water. The *n* and *k* data of these materials are obtained from^30,31^. It can be seen that, in the wavelength range of interest *Δn*_*max*_ = 0.048. This detection range is not possible with *w*_*opt*_ = 270 nm. So we choose to increase the width slightly from the optimum value so that we can decrease *R*, increase *FSR* and accordingly *Δn*_*max*_. We finally choose to work with *w* = 320 nm and *R* = 7.6 µm resulting in *FSR* in the range of 9.85–12.13 nm with a total of 11 resonances (*N* = 11) and *Δn*_*max*_ = 0.058. Figure 2b shows the MRR sensor and the mode profile of the used strip waveguide. We design the forward coupling r of both the reference and the sensing ring resonators such that they work around the critical coupling condition *r* ≈ *ɑ*, to achieve the maximum possible extinction ratio (ER). The forward coupling is determined by the separation *sep* between the straight and the ring waveguide. In our case, the attenuation coefficient ɑ is in the range of 0.96–0.998 for the sensing ring and 0.9395–0.958 for the reference ring. Hence, we choose *sep* = 500 nm which corresponds to r between 0.95 and 0.996. We finally choose that the medium (cladding) index of the reference arm is *n*_*ref*_ = 1.336 and *k*_*ref*_ = 0.

## Results and discussion

### Single element medium

**Figure 3:**
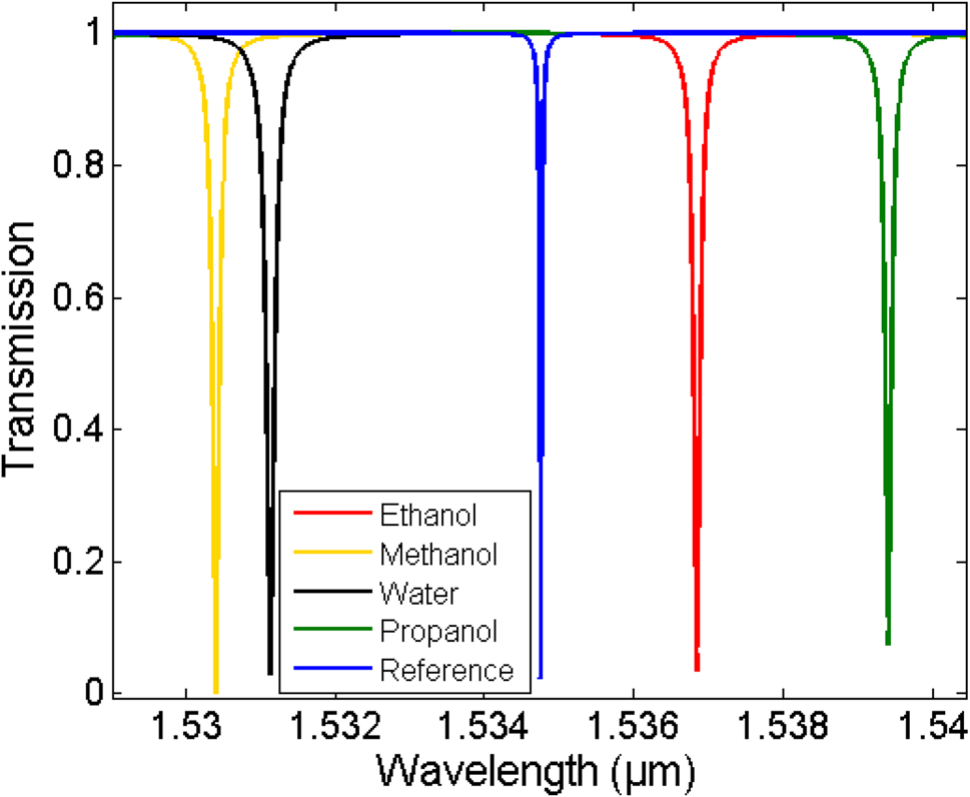
Transmission spectra of the micro-ring resonator at reference and different sensing media.

Here we show how we can selectively detect the substance of a single element medium. The MRR output spectrum of both the reference and the sensing media are determined using mode simulations. Ethanol, methanol, propanol, and water are all used as sensing medium. Finite difference eigenmode (FDE) solver^32^ is used to extract the waveguide complex effective index *n*_*eff*_*(λ)* + *jk*_*eff*_*(λ)* corresponding to the reference and the sensing medium using the *n* and *k* data of the different elements over the wavelength range 1.46–1.6 µm. Then, the output transmission is determined using Eq. (1). This data represents the measured spectrum of the sensor. Figure 3 shows the ring resonator transmission spectra of the reference and the different sensing media namely, ethanol, methanol, propanol, and water. A resolution of 5 pm is used for the transmission spectra which correspond to the spectrometer resolution.

**Figure 4:**
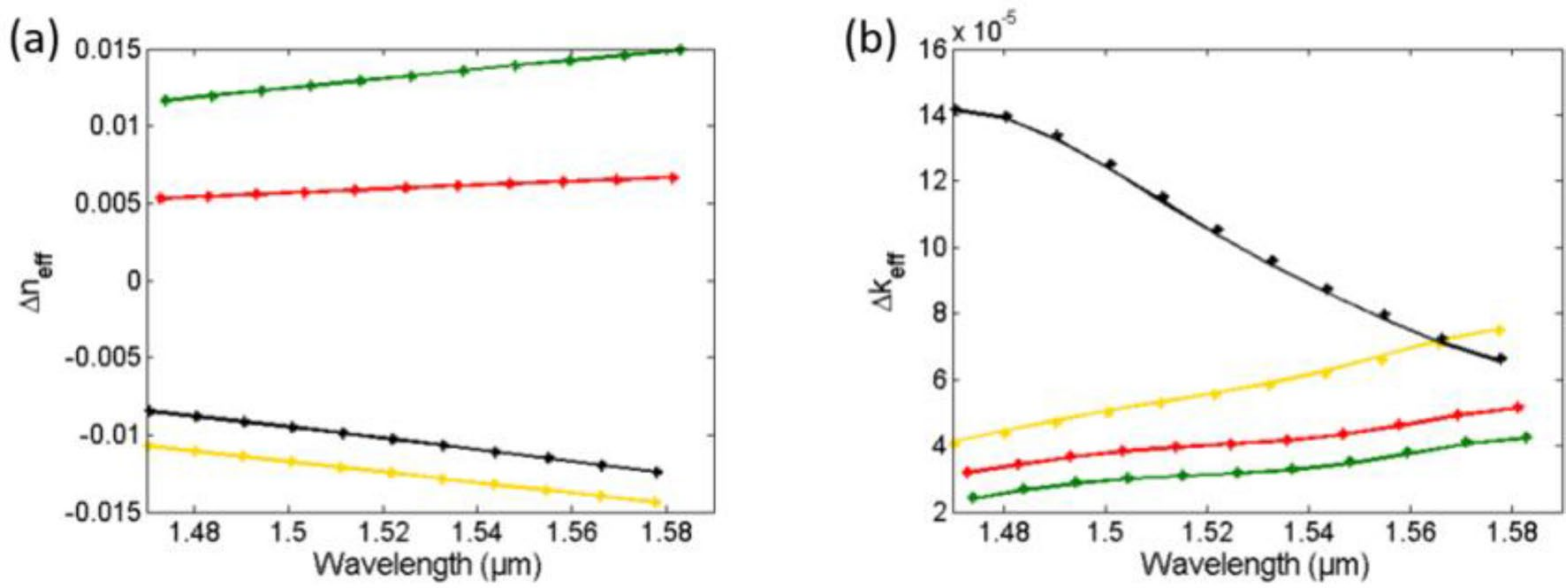
Actual "solid" (from FDE simulation) and detected "asterisk" (from our method) mode effective index change versus wavelength for different sensing media (**a**) Real part Δn_eff_ and (**b**) Imaginary part Δk_eff_.

**Figure 5:**
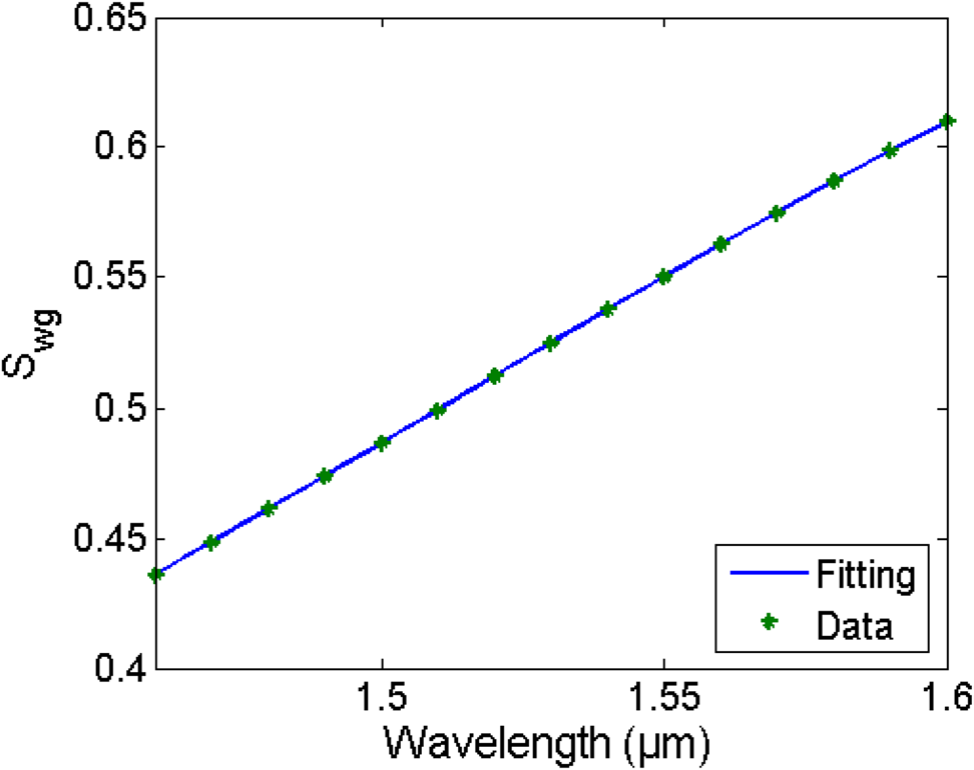
S_wg_ of the fundamental quasi-TE mode versus wavelength of the used SOI waveguide with w = 320 nm and its 4th degree polynomial fitting.

Now we use the method described in the previous section to retrieve *n*_*med,i*_ and *k*_*med,i*_ from these spectra. We first fit each resonance of both the reference and the sensing media with Eq. (1) then get *Δλ*_*res,i*_ and *Δα*_*i*_ from the spectra of Fig. 3. Then, we calculate the change in the waveguide mode effective index *Δn*_*eff,i*_ and *Δk*_*eff,i*_ using Eqs. (6a, 6b). The average fitting accuracy for the different resonances (*N* = 11) is almost 100. Figure 4 shows *Δn*_*eff*_ and *Δk*_*eff*_ for the different sensing media as given from the mode simulations (actual or input) and as calculated from the transmission spectra after resonances fitting (our method). Table 1 shows the average error (for the different resonances) in the detected *Δn*_*eff*_ and *Δk*_*eff*_ for the different substances, showing a maximum error of only 0.053% and 1.23%, respectively. Next, we determined the waveguide sensitivity *S*_*wg*_ = *dn*_*eff*_/*dn*_*med*_ = *dk*_*eff*_/*dk*_*med*_ for our *w* × *h* = 320 nm × 220 nm SOI waveguide using FDE mode simulations. Figure 5 shows the S_wg_ dependence with the wavelength of the fundamental quasi-TE mode and its fitting with a 4th-degree polynomial from 1.46 to 1.6 µm.

**Table 1:** The average error in the detected mode effective index change both real Δn_eff_ and imaginary Δk_eff_ for different sensing media.

	Ethanol	Methanol	Propanol	Water
*Δn*_*eff*_ average error (%)	0.048	0.048	0.053	0.047
*Δk*_*eff*_ average error (%)	0.4	1.23	1.13	1.17

**Figure 6:**
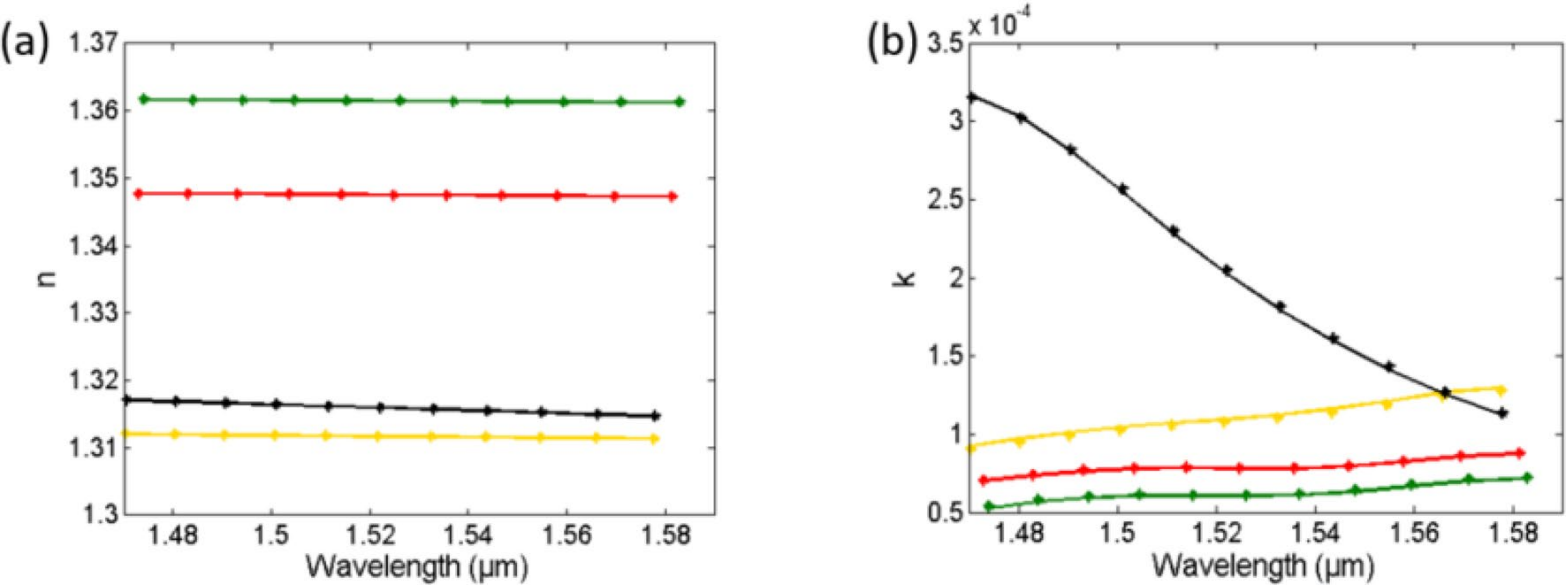
Actual "solid" (from data in^30,31^) and detected "asterisk" (from our method) medium refractive index versus wavelength for different sensing media (**a**) Real part n and (**b**) Imaginary part k.

Finally, we calculate the medium refractive index *n*_*med,i*_ and *k*_*med,i*_ from *Δn*_*eff,i*_ and *Δk*_*eff,i*_ using Eqs. (11a), (11b), (12a) and (12b), knowing that the reference medium is *n*_*ref*_ = 1.336 and *k*_*ref*_ = 0. Figure 6 shows the input (actual) to the sensor medium refractive index data *n* and *k* of the different media, from^30,31^, and the detected values from the transmission spectra using our method. Table 2 shows the average error in the detected *n* and *k* for the different substances, showing a maximum error of only 0.0047% and 1.65%, respectively. All numerical calculations and fitting are done using MATLAB.

**Table 2:** The average error in the detected medium refractive index both real n and imaginary k for different sensing media.

	Ethanol	Methanol	Propanol	Water
*n*_*med*_ average error (%)	0.0004	0.0047	0.0025	0.0033
*k*_*med*_ average error (%)	0.55	1.65	1.48	0.97

### Multi-element medium

In this section, we show how we can determine the concentration composition of a multi-element medium using the same MRR. The complex refractive index of a multi-element medium *n*_*med*_*(λ*_*i*_*)* + *jk*_*med*_*(λ*_*i*_*)* at each wavelength *λ*_*i*_ can be expressed as the linear combination of the different element refractive index *n*_*j*_*(λ*_*i*_*)* + *jk*_*j*_*(λ*_*i*_*)* as follows:

13a $$n_{med} (\lambda_{i} ) = \sum\limits_{j = 1 - M} {c_{j} } * n_{j} (\lambda_{i} )$$

13b $$k_{med} (\lambda_{i} ) = \sum\limits_{j = 1 - M} {c_{j} } * k_{j} (\lambda_{i} )$$

where i = 1 − N the wavelength index with N the number of detected n and k points with wavelength (number of resonances), j = 1 − M the element index with M the number of elements present in the medium and *c*_*j*_ the concentration of each element. Hence, to get the concentration composition *c*_*j*_ of the medium we solve the 2 N linear system of equations Ac = B where A is 2 N × M matrix, B is 2 N × 1 matrix and c is M × 1 matrix as shown:

14a $$A = \left[ {\begin{array}{*{20}l} {n_{1} (\lambda_{1} )} & {n_{2} (\lambda_{1} )} & \cdots & {n_{M} (\lambda_{1} )} \\ {n_{1} (\lambda_{2} )} & {n_{2} (\lambda_{2} )} & \cdots & {n_{M} (\lambda_{2} )} \\ \begin{gathered} \vdots \hfill \\ n_{1} (\lambda_{N} ) \hfill \\ \end{gathered} & \begin{gathered} \vdots \hfill \\ n_{2} (\lambda_{N} ) \hfill \\ \end{gathered} & \begin{gathered} \vdots \hfill \\ \cdots \hfill \\ \end{gathered} & \begin{gathered} \vdots \hfill \\ n_{M} (\lambda_{N} ) \hfill \\ \end{gathered} \\ {k_{1} (\lambda_{1} )} & {k_{2} (\lambda_{1} )} & \cdots & {k_{M} (\lambda_{1} )} \\ {k_{1} (\lambda_{2} )} & {k_{2} (\lambda_{2} )} & \cdots & {k_{M} (\lambda_{2} )} \\ \begin{gathered} \vdots \hfill \\ k_{1} (\lambda_{N} ) \hfill \\ \end{gathered} & \begin{gathered} \vdots \hfill \\ k_{2} (\lambda_{N} ) \hfill \\ \end{gathered} & \begin{gathered} \vdots \hfill \\ \cdots \hfill \\ \end{gathered} & \begin{gathered} \vdots \hfill \\ k_{M} (\lambda_{N} ) \hfill \\ \end{gathered} \\ \end{array} } \right],$$

14b $$B = \left[ {\begin{array}{*{20}l} {n_{med} (\lambda_{1} )} \\ {n_{med} (\lambda_{2} )} \\ \vdots \\ {n_{med} (\lambda_{N} )} \\ {k_{med} (\lambda_{1} )} \\ \begin{gathered} k_{med} (\lambda_{2} ) \hfill \\ \begin{array}{*{20}l} \vdots \\ {k_{med} (\lambda_{N} )} \\ \end{array} \hfill \\ \end{gathered} \\ \end{array} } \right]$$

and

14c $$c = \left[ {\begin{array}{*{20}l} {c_{1} } \\ {c_{2} } \\ \cdots \\ {c_{M} } \\ \end{array} } \right]$$

We tested different media with different concentration compositions of the same four elements; ethanol, methanol, propanol and water. In this case N = 11 and M = 4. Again, a FDE solver is used to determine the complex effective index of the multi-element medium using the medium refractive index calculated from the n and k data of the different elements according to their concentration. Then, we get the corresponding ring-resonator transmission spectrum. To get the matrix c, we first determine the *n*_*med,i*_ and *k*_*med,i*_ from the ring resonator transmission spectra similar to the method used in the previous section which gives the matrix B. Then, using the elements database^30,31^ we have the matrix A. Finally, we use the least square method to determine concentration matrix *c* with the constraints that Σ*c*_*j*_ = 1 and *c*_*j*_ takes values from 0 to 1.

Note that, the determined *n*_*med,i*_ and *k*_*med,i*_ will have errors from two different sources, numerical error and noise error, that will cause inaccuracy in the determined *c*. Numerical errors are due to the different calculations performed to determine *n*_*med,i*_ and *k*_*med,i*_ such as fitting and equation solving. While noise errors in silicon photonic devices arise mainly from temperature fluctuations due to silicon’s high thermo-optic coefficient of 1.8 × 10^–4^ K^−1^. Thus, changes in the temperature will change silicon’s refractive index, as well as other materials’ index, and accordingly will change the effective index of the propagating mode. This will result in a shift in the resonance wavelength which is not due to medium index change causing an error in the determined *n*_*med,i*_ and *k*_*med,i*_. Thus, solving the system of equations with N > M (number of equations greater than number of unknowns) will help minimize the error in the detected concentrations as the least square method will give the matrix *c* that best fits the 2 N equations. Also note that, Eqs. (13a) and (13b) form a set of independent linear equations and hence the least square solution of *Ac* = *B* will be unique as long as N ≥ M.

**Figure 7:**
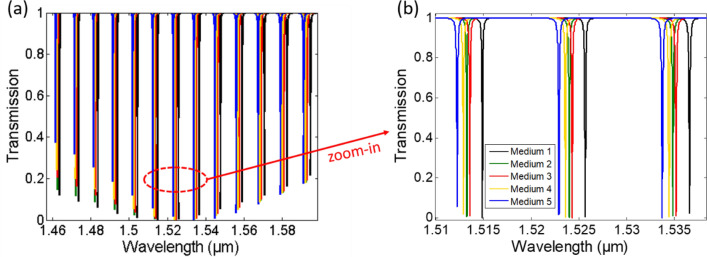
Transmission spectra of the five multi-element media mentioned in Table 3 (**a**) whole spectrum and (**b**) zoomed-in spectrum.

Table 3 shows the actual (input to the sensor) concentrations for five different multi-element media and the detected concentrations using our proposed method as well as the percentage error for the different media. It can be seen that, an error of less than 6.32% is achieved with an average error of 2.6%. Figure 7 shows the transmission spectra corresponding to these five media.

**Table 3:** Actual (input) and detected (using our method) concentration composition for different multi-element media as well as the percentage error. c_W_/e_W_, c_M_/e_M_, c_E_/e_E_ and c_P/_/e_P_ are the water, methanol, ethanol and propanol concentrations/error, respectively.

	Medium 1	Medium 2	Medium 3	Medium 4	Medium 5
Actual	*c*_*W*_ = 0.1	*c*_*W*_ = 0.1	*c*_*W*_ = 0.2	*c*_*W*_ = 0.25	*c*_*W*_ = 0.5
	*c*_*M*_ = 0.15	*c*_*M*_ = 0.3	*c*_*M*_ = 0.2	*c*_*M*_ = 0.25	*c*_*M*_ = 0.1
	*c*_*E*_ = 0.25	*c*_*E*_ = 0.4	*c*_*E*_ = 0.3	*c*_*E*_ = 0.25	c_E_ = 0.25
	*c*_*P*_ = 0.5	*c*_*P*_ = 0.2	*c*_*P*_ = 0.3	*c*_*P*_ = 0.25	*c*_*P*_ = 0.15
Detected	*c*_*W*_ = 0.1	*c*_*W*_ = 0.1	*c*_*W*_ = 0.2	*c*_*W*_ = 0.25	*c*_*W*_ = 0.5
	*c*_*M*_ = 0.15	*c*_*M*_ = 0.3	*c*_*M*_ = 0.2	*c*_*M*_ = 0.25	*c*_*M*_ = 0.1
	*c*_*E*_ = 0.24	*c*_*E*_ = 0.42	*c*_*E*_ = 0.29	*c*_*E*_ = 0.26	*c*_*E*_ = 0.26
	*c*_*P*_ = 0.51	*c*_*P*_ = 0.19	*c*_*P*_ = 0.31	*c*_*P*_ = 0.25	*c*_*P*_ = 0.14
Error (%)	*e*_*W*_ = 0.56	*e*_*W*_ = 0.2	*e*_*W*_ = 0.4	*e*_*W*_ = 0.04	*e*_*W*_ = 0.33
	*e*_*M*_ = 2.45	*e*_*M*_ = 1.67	*e*_*M*_ = 1.58	*e*_*M*_ = 0.72	*e*_*M*_ = 5.28
	*e*_*E*_ = 4.59	*e*_*E*_ = 4.64	*e*_*E*_ = 2.86	*e*_*E*_ = 2.67	*e*_*E*_ = 5.25
	*e*_*P*_ = 1.67	*e*_*P*_ = 6.68	*e*_*P*_ = 2.08	*e*_*P*_ = 1.91	*e*_*P*_ = 6.32

The same design principle can be used to detect other substances for different applications. We only need to design the MRR properly (as described in the “MRR sensor design” subsection) such that it can detect the range of *n* and *k* of all the substances present in the medium along a certain wavelength range. Note that, unknown substances cannot be detected in this case. However, we can still determine the concentrations of the known substances with a reasonable error if this unknown substance is of low concentration. In this case, the unknown substance will contribute to an additional error in the determined *n*_*med,i*_ and *k*_*med,i*_. But again the least square method with N > M will still give us the concentration matrix *c* that best fits *n*_*med,i*_ and *k*_*med,i*_. Hence, for accurate detection of the substances concentrations, we need to know the different substances that may be present in the medium even with small concentrations.

According to this discussion, we can say that our proposed method is promising for selective detection without the need for a complex surface functionalization process. Surface functionalization is used to selectively detect a certain substance that is present in a complex (mullti-element) medium. So, by providing the *n* and *k* data for all the possible elements that may be present in the medium for certain application our method can be used to determine the presence of a specific substance in this medium.

## Conclusion and future work

We propose a novel technique for selective on-chip optical detection. It is based on determining the complex refractive index *n* + *jk* of the medium at multiple wavelengths using a MRR cavity. Using this technique the substance present in the sample can be specified by the refractive index dispersion of the medium. It can also detect the concentration composition of a multi-element sample using a single measurement. This technique can be used even with substances that have a low absorption coefficient and it may overcome the need for surface functionalization process. Our technique was demonstrated using methanol, ethanol, propanol, and water as medium substances. High accuracy in detecting both *n* and *k* has been achieved. We aim to empower our method capabilities through artificial intelligence (AI) algorithms in future work. Using AI algorithms we can reach even higher detection accuracy, detect more complex media and detect changes in temperature and other parameters.

## Data Availability

The datasets used and/or analysed during the current study available from the corresponding author on reasonable request.
